# Translating SGRT from Breast to Lung Cancer: A Study on Frameless Immobilization and Real-Time Monitoring Efficacy, Focusing on Setup Accuracy

**DOI:** 10.3390/life15081234

**Published:** 2025-08-04

**Authors:** Jang Bo Shim, Hakyoung Kim, Sun Myung Kim, Dae Sik Yang

**Affiliations:** 1 Departments of Radiation Oncology, Korea University Guro Hospital, Korea University College of Medicine, Seoul 02841, Republic of Korea; jjankola@hanmail.net (J.B.S.); sunmyung01@hanmail.net (S.M.K.); 2 Departments of Medical Physics, Kyonggi University, Seoul 16227, Republic of Korea

**Keywords:** surface-guided radiotherapy, lung cancer, frameless, accuracy

## Abstract

**Objectives:** Surface-Guided Radiation Therapy (SGRT) has been widely adopted in breast cancer radiotherapy, particularly for improving setup accuracy and motion management. Recently, its application in lung cancer has attracted growing interest due to similar needs for precision. This study investigates the feasibility and clinical utility of SGRT in lung cancer treatment, focusing on its effectiveness in patient setup and real-time motion monitoring under frameless immobilization conditions. **Materials and Methods:** A total of 204 treatment records from 17 patients with primary lung cancer who underwent radiotherapy at Korea University Guro Hospital between October 2024 and April 2025 were retrospectively analyzed. Patients were initially positioned using the Identify system (Varian) in the CT suite, with surface data transferred to the treatment room system. Alignment was performed to within ±1 cm and ±2° across six degrees of freedom. Cone-beam CT (CBCT) was acquired prior to treatment for verification, and treatment commenced when the Distance to Correspondence Surface (DCS) was ≤0.90. Setup deviations from the Identify system were recorded and compared with CBCT in three translational axes to evaluate positioning accuracy and PTV displacement. **Results and Conclusions:** The Identify system was shown to provide high setup accuracy and reliable real-time motion monitoring in lung cancer radiotherapy. Its ability to detect patient movement and automatically interrupt beam delivery contributes to enhanced treatment safety and precision. In addition, even though the maximum longitudinal (Lng) shift reached up to −1.83 cm with surface-guided setup, and up to 1.78 cm (Lat) 5.26 cm (Lng), 9.16 cm (Vrt) with CBCT-based verification, the use of Identify’s auto-interruption mode (±1 cm in translational axes, ±2° in rotational axes) allowed treatment delivery with PTV motion constrained within ±0.02 cm. These results suggest that, due to significant motion in the longitudinal direction, appropriate PTV margins should be considered during treatment planning. The Identify system enhances setup accuracy in lung cancer patients using a surface-guided approach and enables real-time tracking of intra-fractional errors. SGRT, when implemented with systems such as Identify, shows promise as a feasible alternative or complement to conventional IGRT in selected lung cancer cases. Further studies with larger patient cohorts and diverse clinical settings are warranted to validate these findings.

## 1. Introduction

Accurate patient setup and reproducibility are critical components in radiation therapy, particularly with tumors such as those in breast or lung cancer, which exhibit motion of the chest wall or lung parenchyma due to respiration, where even minor variations in positioning can significantly affect dose distribution and clinical outcomes. Conventional setup techniques typically rely on permanent skin tattoos and laser alignment systems to ensure proper patient positioning. However, these methods are associated with several limitations, including limited reproducibility, increased setup time, and patient discomfort or dissatisfaction related to the permanent markings.

Surface-Guided Radiation Therapy (SGRT) has emerged as a promising non-invasive alternative that addresses many of the shortcomings of conventional techniques [1,2]. SGRT utilizes advanced optical surface imaging technology to continuously monitor the patient’s external anatomy in real time. By capturing three-dimensional surface data, SGRT enables accurate patient positioning and delivers six-degree-of-freedom (6DoF) corrections, encompassing translational and rotational adjustments across all axes. This high-resolution tracking capability facilitates not only precise initial setup but also effective intra-fractional motion management throughout the course of treatment. As a result, SGRT offers a non-invasive, marker-free approach to maintaining spatial accuracy, reducing the need for frequent imaging, and potentially minimizing patient exposure to additional radiation. Its integration into modern radiotherapy workflows represents a significant advancement in ensuring treatment fidelity, particularly in anatomical sites subject to respiratory motion, such as the thorax. This technology has proven particularly valuable in the implementation of Deep Inspiration Breath Hold (DIBH) protocols, where consistent and reproducible control of respiratory motion is essential. DIBH is commonly employed to reduce radiation exposure to adjacent critical structures, and its clinical relevance is especially well-established in the treatment of left-sided breast cancer, where minimizing cardiac dose is a primary concern. By enabling real-time monitoring of the patient’s thoracic surface during breath-hold, SGRT enhances the accuracy and reproducibility of breath-hold positioning across treatment fractions. This capability not only improves target localization but also contributes to the overall safety and effectiveness of radiation delivery. As a result, SGRT has become a key component in modern DIBH workflows, supporting efforts to improve both oncologic outcomes and long-term cardiac health in breast cancer patients [3,4,5,6,7,8,9,10,11]. Beyond its technical advantages, SGRT enables tattoo-free treatment workflows, eliminating the need for permanent skin markings and thereby improving patient experience and psychological well-being. Moreover, SGRT systems are capable of automatically interrupting radiation delivery in the event of patient movement, enhancing treatment safety and providing additional quality assurance measures throughout the treatment process.

Recent studies have begun to explore the application of SGRT in lung cancer patients, showing promising results in improving setup accuracy and reducing positioning errors compared to conventional methods [1,2,12,13,14,15]. As evidence continues to accumulate, SGRT has the potential to enhance the quality of care in thoracic radiotherapy. This study aims to evaluate the feasibility and clinical utility of applying SGRT—a technique widely adopted in breast cancer treatment—to lung cancer patients. Specifically, we assess its effectiveness in ensuring accurate initial patient setup and reliable real-time motion monitoring within a frameless immobilization setting. Given the challenges posed by respiratory motion and complex thoracic anatomy, this study explores whether the precision and workflow efficiency achieved in breast radiotherapy can be extended to lung cancer treatment without relying on rigid immobilization devices.

## 2. Materials and Methods

### 2.1. Patients

A total of 204 treatment records from 17 patients with primary lung cancer who underwent radiotherapy at Korea University Guro Hospital between October 2024 and April 2025 were retrospectively analyzed. All patients included in this study were diagnosed with primary non-small cell lung cancer (NSCLC) and received treatment exclusively for this primary lung lesion. In addition, all patients provided informed consent for the use of non-reimbursed SGRT. Detailed information on patient demographics and radiation treatment planning parameters—including the fractionation scheme, total dose, radiotherapy technique, and clinical target volume (CTV)—is presented in Table 1. This study was approved by the Institutional Review Board (IRB) of Korea University Guro Hospital (IRB No. 2023GR0216), and the requirement for informed consent was waived due to the retrospective nature of the study.

### 2.2. Treatment Scheme and Planning

The planned total dose and number of fractions varied according to the treatment intent and the location of the primary lung lesions (Table 1). In accordance with institutional guidelines, seven patients with small (≤4 cm), peripherally located NSCLC lesions were treated with stereotactic ablative radiotherapy (SABR), receiving a total dose of 60 Gy over 4 to 5 fractions. An additional eight patients underwent conventional fractionated radiotherapy with a total dose of 60 Gy administered in 20 fractions, while the remaining two patients received 30 Gy in 10 fractions.

For target delineation of lung lesions in IMRT planning, the gross tumor volume (GTV) was delineated under the lung window settings. The internal target volume (ITV) was delineated following four-dimensional CT with special regard to the patient’s respiratory motion. The clinical target volume (CTV) was generated with a 5 mm expansion of the GTV-ITV in all directions and then modified according to the adjacent normal anatomic structures. The planning target volume (PTV) was generated with a 5 mm expansion of the CTV. The prescription guideline was to deliver at least 97% of the prescribed dose to 95% of the PTV. The minimum and maximum doses to 1cc of PTV were 95% and 107%, respectively.

For target delineation of lung lesions in SBRT planning, the GTV was delineated under the lung window settings. The GTV-ITV was delineated following four-dimensional CT with special regard to the patient’s respiratory motion. The PTV was generated with a 5 mm expansion of the GTV-ITV. The prescription guideline was that 95% of PTV should be covered by prescription dose. The percentage lung volume that received ≥20 Gy was to be kept ≤35%, and the mean lung dose was ≤20 Gy. The maximum doses to the spinal cord and esophagus were not to exceed 45 Gy and 60 Gy, respectively, satisfying the dose-volume constraints of normal organ.

Each patient was immobilized on a shoulder board, and fiducial markers (lead beads) were placed near the tumor site to aid in localization. Surface imaging was performed in the CT suite using the Identify system (Varian). Following free-breathing practice, CT scans were acquired with a slice thickness of 2 mm for SABR plans and 3 mm for Volumetric Modulated Arc Therapy (VMAT) or Intensity Modulated Radiation Therapy (IMRT) plans. Treatment planning was performed using the Eclipse Treatment Planning System version 16.1 (Varian). VMAT with 2 to 4 arcs was used for most patients. For Patient 14, IMRT with 5 portals and 830 segments was employed. The treatment plan was calculated using the Anisotropic Analytical Algorithm (AAA) with a calculation resolution of 0.25 cm, employing a 6 MV FFF beam at a dose rate of 1200 MU/min. The role of the fiducial markers was limited to indicating the origin point during CT simulation and marking the initial original center in the radiation treatment plan. The fiducial markers were not used during the actual treatment.

### 2.3. Setup Before Treatment and Analysis

The patient’s treatment plan, including the reference surface contours generated during simulation, was imported into the Identify system (Varian Medical Systems) located in the treatment room. Using the system’s real-time optical surface tracking capability, the patient was initially positioned on the treatment couch such that the external surface at the treatment site aligned with the planned surface contours within a tolerance of ±1 cm in translational axes (vertical [Vrt], longitudinal [Lng], lateral [Lat]) and ±2° in rotational axes (pitch, roll, rotation [Rtn]), covering all six degrees of freedom. This initial alignment ensured that the patient’s position corresponded closely to the planned setup parameters prior to imaging verification. Following this surface-guided setup, a cone-beam computed tomography (CBCT) scan was acquired with the imaging volume centered on the PTV to verify internal anatomy and confirm target localization. Adjustments were made if necessary to reconcile any discrepancies between external surface alignment and internal target position. Once the CBCT-based alignment was confirmed, treatment was initiated when the Distance to Correspondence Surface (DCS)—a quantitative measure of deviation between the real-time patient surface and the planned reference surface—fell within the clinically accepted threshold of 0.90. This ensured that both external and internal anatomical landmarks were accurately aligned before radiation delivery commenced.

The Dice Similarity Coefficient (DSC), or Dice coefficient, is a measure of similarity between two sets. It is commonly used in medical image processing and segmentation evaluation.

$$DSC=\frac{2|A\cap B|}{\left|A\right|+\left|B\right|}$$

Let |A| be the number of elements in set A, |B| be the number of elements in set B, and |A ∩ B| be the number of elements common to both A and B. A value closer to 1 indicates a high degree of overlap or agreement between the two sets, while a value closer to 0 indicates greater dissimilarity.

Pre-treatment setup values obtained from the Identify system were systematically recorded across all six degrees of freedom, encompassing three translational (vertical [Vrt], longitudinal [Lng], lateral [Lat]) and three rotational (pitch, roll, rotation) axes. To assess the accuracy of surface-guided positioning, these recorded values were compared against CBCT images, which served as the reference standard for internal anatomical alignment. Positioning errors were specifically evaluated along the three translational axes by quantifying discrepancies between the Identify system measurements and CBCT-based target localization. Based on these comparisons, displacements of the PTV were analyzed to determine the precision and reliability of SGRT-based setup in reproducing the intended treatment position.

## 3. Results

**Figure 1:**
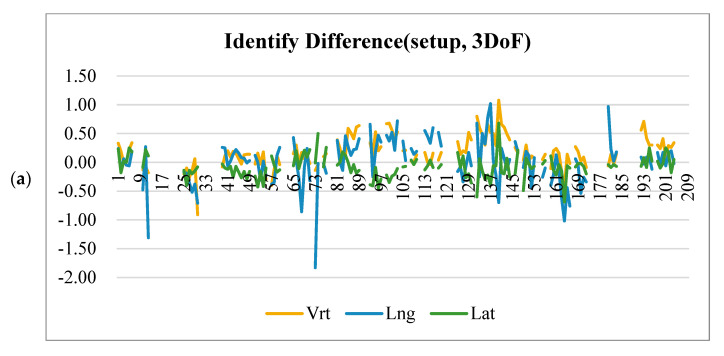

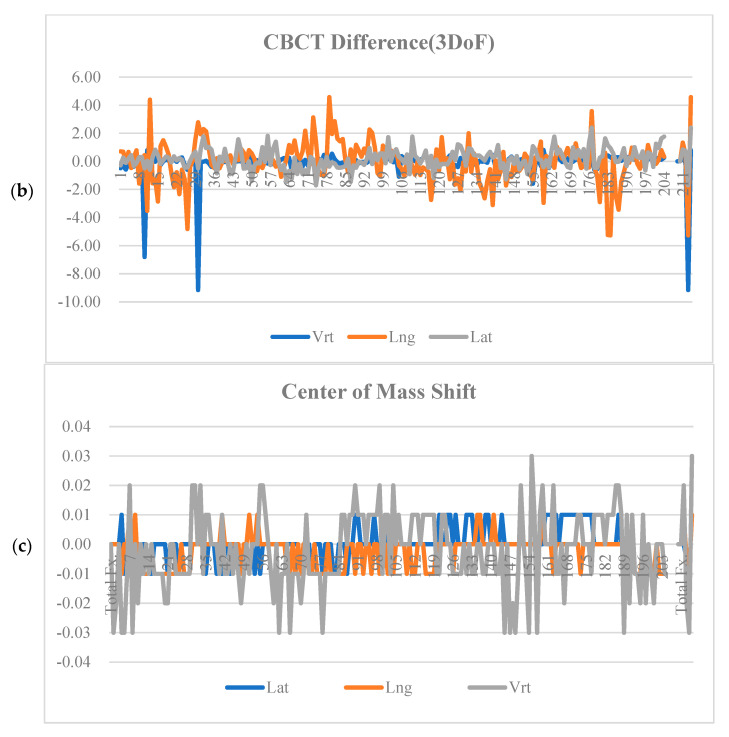
Intra-fractional shifts in the patient by Identify (**a**), shift in the patient imaging (**b**) and the tumor (**c**) for each individual patient in the vertical (vrt), longitudinal (lng), and lateral (lat) directions.

Using surface-guided setup with the Identify system, setup errors verified by CBCT were analyzed over a total of 204 treatment fractions. The mean setup errors observed were ±0.71 cm in the vertical (Vrt) axis, ±0.78 cm in the longitudinal (Lng) axis, and ±0.45 cm in the lateral (Lat) axis, demonstrating generally small deviations from the planned treatment position. Upon acquisition of CBCT images, residual setup errors were further refined, with values of ±0.22 cm (Vrt), ±0.68 cm (Lng), and ±0.57 cm (Lat) recorded, indicating a reduction in positioning uncertainty following image-guided corrections. Displacement analysis of the CTV revealed minimal shifts, with average displacements of ±0.01 cm in both the vertical and longitudinal directions and no measurable displacement in the lateral axis. These findings suggest a high degree of stability in target localization when using SGRT combined with CBCT verification. Furthermore, the spatial concordance between planned and delivered target volumes was quantified using the Dice Similarity Coefficient (DSC), which yielded values of ≥0.91 for the CTV across all fractions. This high DSC value reflects excellent overlap and agreement between the planned target contours and the actual anatomical position during treatment (Figure 1), underscoring the accuracy and reliability of the combined surface-guided and image-guided setup approach.

In the setup using the Identify system, the maximum error occurred in the longitudinal (Lng) direction with a value of 1.83 cm, while in the CBCT images, the largest error was observed in the vertical (Vrt) direction at 9.16 cm. The maximum displacement of the CTV was only 0.03 cm in the vertical direction. In the Identify setup, larger errors occurred in the Vrt and Lng directions compared to the lateral (Lat) direction, and a similar pattern was observed in the CBCT images. Notably, the largest center of mass shift was also observed in the Vrt direction.

**Figure 2:**
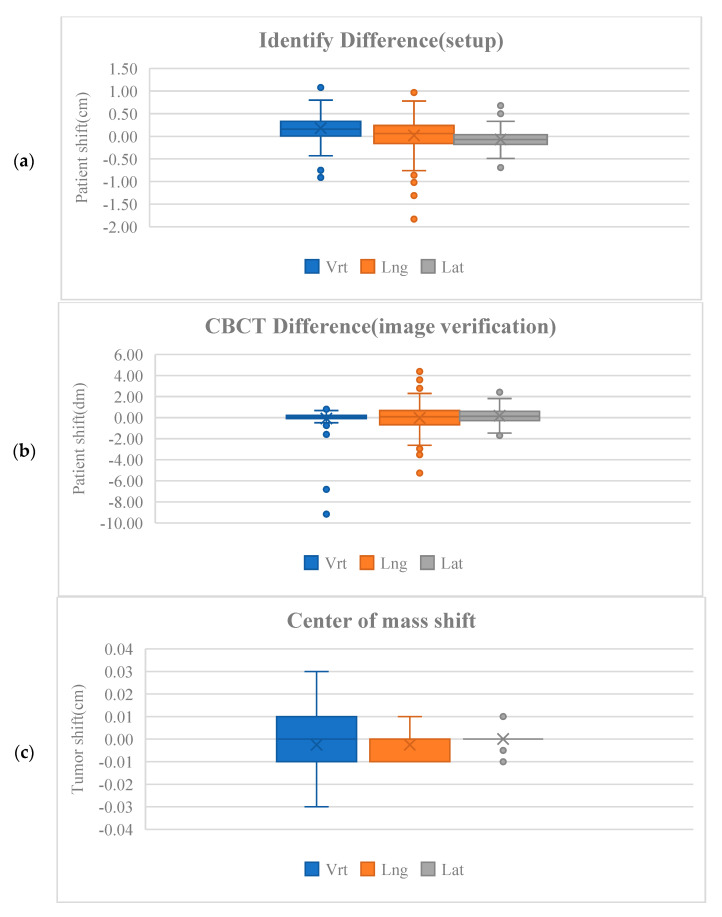
Box and whisker plots for the intra-fractional shifts in the patient by identify (**a**), shift in the patient imaging (**b**) and the tumor (**c**) for the 204 fractions studied. The cross represents the mean value and the middle line in the box represents the median value.

The median values of the surface-guided setup measurements obtained from the Identify system during treatment were 0.34 cm in the vertical (Vrt) axis, 0.11 cm in the longitudinal (Lng) axis, and 0.14 cm in the lateral (Lat) axis. The corresponding interquartile ranges (IQRs) were 0.288 cm, 0.400 cm, and 0.200 cm, respectively, indicating relatively consistent positioning accuracy with limited variability across treatment fractions. In comparison, the median setup deviations assessed via CBCT during treatment were −0.12 cm (Vrt), 0.52 cm (Lng), and 0.74 cm (Lat), with wider IQRs of 0.285 cm, 1.338 cm, and 0.845 cm, respectively. These results suggest that while CBCT detected slightly larger deviations in patient positioning, particularly in the longitudinal and lateral axes, the overall setup remained within clinically acceptable limits. Analysis of PTV shifts revealed minimal median displacements of −0.02 cm (Vrt), −0.01 cm (Lng), and −0.01 cm (Lat), with very narrow IQRs of 0.020 cm, 0.010 cm, and 0.000 cm, respectively. These findings indicate excellent stability of the target volume throughout treatment sessions, reinforcing the effectiveness of the combined surface-guided and image-guided setup protocols. Detailed data distributions are illustrated in Figure 2.

## 4. Discussion

While SGRT has become a well-established technique in breast cancer radiotherapy—DIBH approaches aimed at minimizing cardiac exposure—its application in lung cancer treatment remains relatively limited. In breast cancer, SGRT has been shown to improve treatment accuracy by enabling precise patient positioning and real-time monitoring of respiratory motion, significantly reducing heart dose and thereby decreasing the risk of radiation-induced cardiac toxicity [3,4,5,6,7,8,9,10,11]. However, the adoption of SGRT in lung cancer has faced challenges due to the complexity of respiratory motion and tumor heterogeneity within the thoracic cavity, as well as difficulties in correlating surface motion with internal tumor. Recent studies suggest that while SGRT offers potential advantages in managing intrafractional motion during lung cancer radiotherapy, its clinical integration requires further validation through larger trials and development of robust motion correlation algorithms [16,17]. Therefore, despite promising initial results, SGRT’s role in lung cancer remains an active area of investigation.

Recently, the implementation of SGRT technologies, with AlignRT being a prominent example, has been associated with notable improvements in the spatial accuracy and precision of SABR delivery in patients with lung cancer. Hai-Liang Guo [13] emphasizes that SGRT can effectively monitor patient positioning both before and during treatment, providing a reliable means of adjusting treatment margins with greater precision. This approach reduces radiation exposure to surrounding healthy tissues and increases treatment accuracy. Moreover, Guo suggests that defining treatment errors within the 95% probability density interval enhances the overall treatment precision. Prior to this, Sebastian Sarudis [15] highlights that in a study involving 137 segments, more than 96.4% of cases showed patient positioning deviations within 2 mm. Most tumors were successfully maintained within the planned ITV, demonstrating that SGRT, when properly implemented, can achieve positioning accuracy comparable to traditional complex SBRT fixation devices without the need for them. However, in line with the concerns previously raised by Keall [16] and Wang [17], Gavin Lawler [2] raises an important consideration regarding the use of surface guidance (SG) alone. While SG is effective in conjunction with stringent Image Guided Radiation Therapy (IGRT) protocols, reliance on SG without IGRT may lead to discrepancies between surface and internal tumor movements, which requires careful attention.

Most existing studies have primarily concentrated on the use of SGRT in high-dose, hypofractionated regimens such as SABR, where its benefits in enhancing setup accuracy and minimizing geometric uncertainties are well-documented. However, the role of SGRT in more conventional fractionation schemes and broader clinical contexts remains relatively underexplored. In this regard, the present study was designed to investigate the feasibility and clinical utility of SGRT in a more diverse lung cancer patient population—specifically, individuals receiving radiotherapy for primary lung lesions across a variety of fractionation protocols, including conventional and moderately hypofractionated regimens. This broader application is of particular interest, as it reflects real-world clinical scenarios that extend beyond the narrow subset of SABR-eligible patients. By assessing SGRT in this wider context, our findings may contribute to expanding its use as a standard positioning and monitoring tool in thoracic radiotherapy. The integration of SGRT in these settings could potentially improve reproducibility of patient setup, enhance inter-fraction consistency, and reduce reliance on frequent imaging, thereby supporting workflow efficiency and patient comfort. Through this study, the Identify system was shown to provide high setup accuracy and reliable real-time motion monitoring in lung cancer radiotherapy. Its ability to detect patient movement and automatically interrupt beam delivery contributes to enhanced treatment safety and precision. In addition, even though the maximum longitudinal (Lng) shift reached up to −1.83 cm with surface-guided setup, and up to 1.78 cm (Lat) 5.26 cm (Lng), 9.16 cm (Vrt) with CBCT-based verification, the use of Identify’s auto-interruption mode (±1 cm in translational axes, ±2° in rotational axes) allowed treatment delivery with PTV motion constrained within ±0.02 cm. These results suggest that, due to significant motion in the longitudinal direction, appropriate PTV margins should be considered during treatment planning. The Identify system enhances setup accuracy in lung cancer patients using a surface-guided approach and enables real-time tracking of intra-fractional errors.

## 5. Conclusions

These findings suggest that SGRT, when integrated with systems like Identify, may serve as a valuable alternative or adjunct to conventional IGRT in selected cases of lung cancer. Further investigation involving larger cohorts and varied clinical scenarios is needed to confirm and expand upon these results.

## Figures and Tables

**Table 1:** Patient demographics and radiation treatment planning parameters.

Number of Patient	Fractionation Scheme	Tumor Location	Total Dose	Technique		CTV Volume (cm^3^)
1	1500/4	RUL	60 Gy	SABR/VMAT	4ARC	39.2
2	1200/5	RUL	60 Gy	SABR/VMAT	4ARC	31.9
3	300/20	RUL	60 Gy	VMAT	2ARC	102.7
4	1200/5	LLL	60 Gy	SABR/VMAT	4ARC	53.4
5	300/20	RUL	60 Gy	VMAT	2ARC	214.1
6	1200/5	RUL	60 Gy	SABR/VMAT	4ARC	42.0
7	1200/5	LUL	60 Gy	SABR/VMAT	4ARC	21.2
8	300/20	RUL	60 Gy	VMAT	2ARC	195.6
9	300/20	RML	60 Gy	VMAT	2ARC	129.7
10	300/20	RLL	60 Gy	VMAT	2ARC	41.2
11	300/20	LUL	60 Gy	VMAT	2ARC	50.1
12	300/10	RML	30 Gy	VMAT	2ARC	61.4
13	1200/5	LUL	60 Gy	SABR/VMAT	4ARC	7.0
14	300/20	LLL	60 Gy	IMRT	5/830	311.4
15	300/10	RUL	30 Gy	VMAT	2ARC	265.9
16	1200/5	RUL	60 Gy	SABR/VMAT	4ARC	22.8
17	300/20	RUL	60 Gy	VMAT	2ARC	89.0

Abbreviations: CTV, clinical target volume; SABR, stereotactic ablative radiation therapy; VMAT, volumetric modulated arc therapy; IMRT, Intensity modulated radiation therapy.

## Data Availability

The datasets used and/or analyzed in the current study can be obtained from the corresponding author upon reasonable request.
